# Rapid detection of Akabane virus by a novel reverse transcription loop-mediated isothermal amplification assay (RT-LAMP)

**DOI:** 10.1186/1743-422X-10-288

**Published:** 2013-09-14

**Authors:** Jun Qiao, Junwei Wang, Qingling Meng, Guochao Wang, Yucheng Liu, Zhihao He, Haibo Yang, Zaichao Zhang, Xuepeng Cai, Chuangfu Chen

**Affiliations:** 1 College of Animal Science and Technology, Shihezi University, Shihezi, Xinjiang 832003, People’s Republic of China; 2 State Key Lab of Veterinary Etiological Biology, Lanzhou Veterinary Research Institute, Chinese Academy of Agricultural Sciences, Lanzhou, Gansu 730046, People’s Republic of China

**Keywords:** Rapid detection, Akabane virus, Reverse transcription loop-mediated isothermal amplification assay

## Abstract

**Background:**

Akabane disease, caused by Akabane virus, is an insect-transmitted disease of ruminants that is primarily characterized by fetal damage.

**Methods and results:**

In this study, a novel reverse transcription loop-mediated isothermal amplification (RT-LAMP) assay for rapid detection of Akabane virus was successfully developed. The primers were designed to target the highly conserved fragment of nucleoprotein from the Akabane virus. The results indicate that the assay is highly specific and sensitive with a detection limit of 5.0 TCID_50_ /mL within a 60-min incubation time. A total of 126 abortive samples collected from Xinjiang province were detected by the established RT-LAMP. The results of RT-LAMP assay showed 96.8% agreement with the semi-nested RT-PCR.

**Conclusion:**

This study is to first to develop a rapid, sensitive, and accurate method for the detection of Akabane virus, which may be used to screen clinical samples in developing countries or regions.

## Background

Akabane virus, the causative agent of enzootic bovine arthrogryposis and hydranencephaly, is an arbovirus in the genus Orthobunyavirus and the Simbu sero-group of the family *Bunyaviridae*. This particular virus is transmitted among animals by hematophagous arthropod vectors such as mosquitoes and culicoides biting midges [1]. The infection of Akabane virus in pregnant cows can lead to a series of endemic and abnormal deliveries such as abortion, premature birth, stillbirth, and birth of abnormal calves [1-5]. Between 1972 to 1975, more than 40,000 cattle were infected in Japan [6], which resulted in a huge economic loss to the cattle industry. To date, Akabane disease is widely prevalent in Japan, Korea, Israel, Central Africa, and Australia [1,4,5,7,8].

Although laboratory diagnostic techniques for Akabane disease such as viral neutralization test (VNT), enzyme-linked immunosorbent assays (ELISAs) [9-12], immunoperoxidase staining [13,14], dot immunobinding assay [15], nested reverse transcription polymerase chain reaction (RT-PCR) [16], multiplex RT-PCR [17] and real-time RT-PCR [18] have been established, most of the current serological tests have cross-reactions with other related viruses, particularly those in the simbu serogroup [9,15,19]. PCR-based molecular techniques need specialized equipment and may not be readily available in diagnostic laboratories of underdeveloped and developed countries [16]. Viral isolation from aborted fetus is rarely successful because of an elicited immune response that inhibits virus replication. Therefore, it is necessary to develop new assays for the detection of Akabane virus in the clinic.

In this study, we demonstrated that loop-mediated isothermal amplification (LAMP) provides a new method for molecular diagnosis [20]. LAMP has been widely used to detect different pathogens because of its rapid detection [21,22]. The aim of this study is to develop a rapid and accurate molecular tool for detecting Akabane virus in clinical specimens.

## Results and discussion

**Figure 1:**
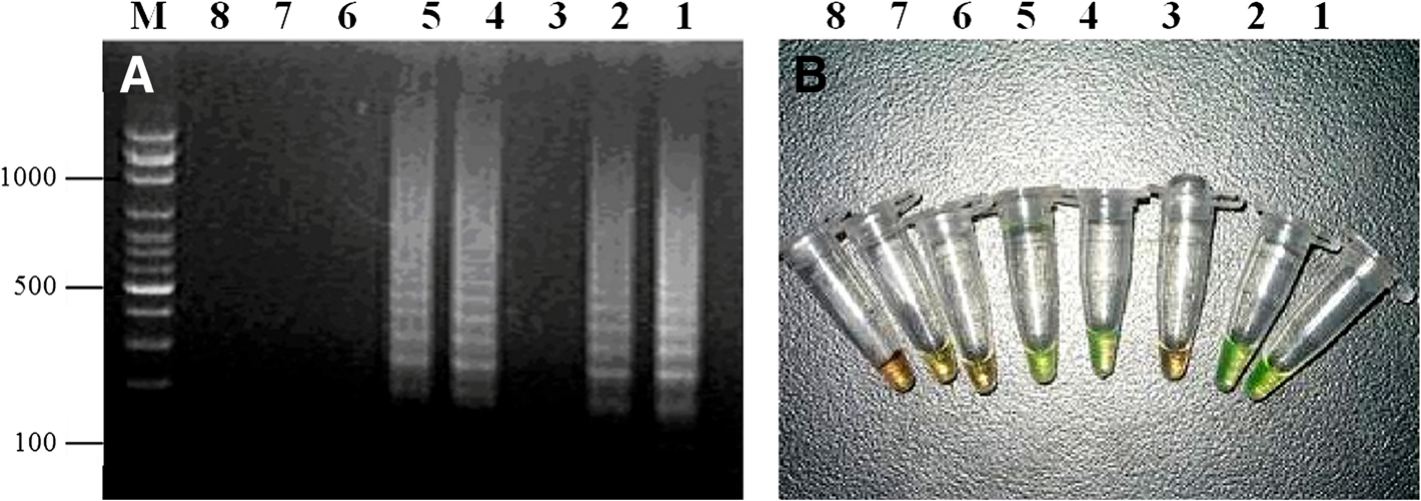
**Specificity of RT-LAMP. A**, Agarose gel electrophoresis showing the specificity of RT-LAMP on amplification of different viruses. **B**, Visual detection of RT-LAMP amplification products of the different viruses using SYBR Green I staining. The color change from orange in the negative to green in the positive reaction. The lane and tube numbers correspond to the following specimens: Lanes 1, 2, 4 and 5, Akabane virus strain JaGAR 39/MB19; Lane 3, *Bovine herpesvirus* 1 strain 758; Lane 6, *Bovine parvovirus* strain BF15; Lane 7, *Bluetongue virus* strain CSIRO 154; Lane 8, *Bovine viral diarrhea virus* strain NADL; Lane M, 100-bp DNA Ladder.

**Figure 2:**
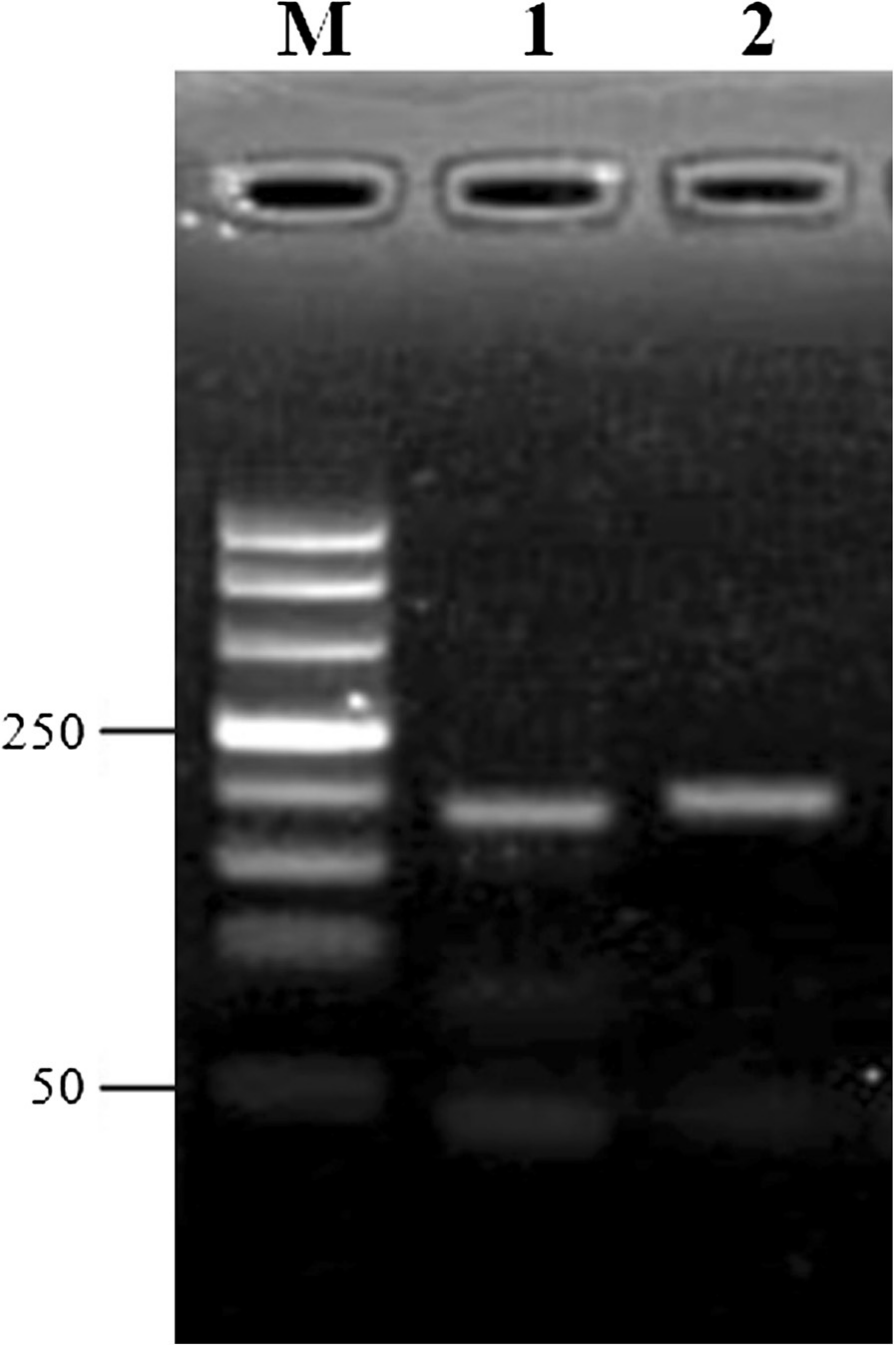
**Identification of the RT-LAMP amplification products by *Acc*III digestion.** The amplification products were digested by *Acc*III, appearing as 2 fragments at 33 and 180 bp in size. Lane M, 50-bp DNA Ladder (500, 400, 300, 250, 200, 150, 100, 50); Lane 1, the amplification products was digested by *Acc*III; Lane 2, the amplification products.

**Figure 3:**
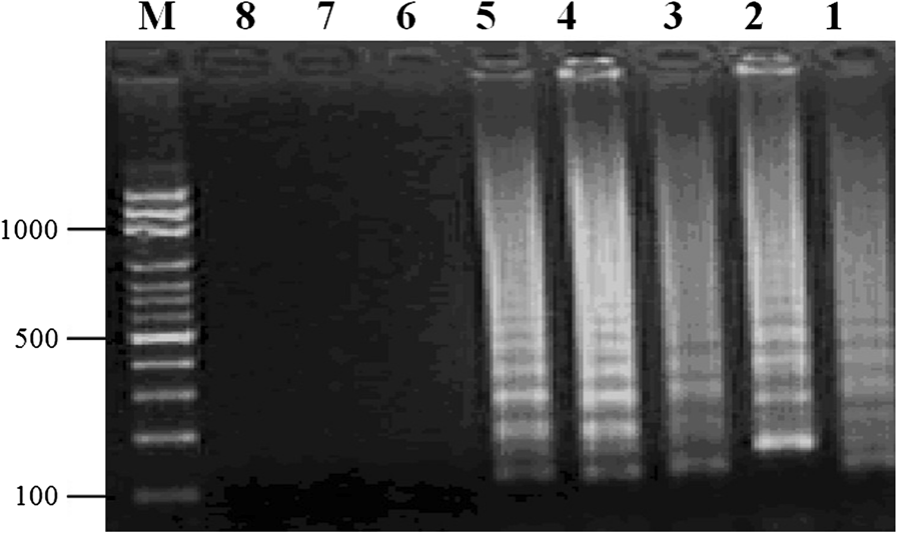
**Sensitivity of RT-LAMP.** The sensitivity of RT-LAMP was determined using 10-fold serial dilutions of the culture supernatant contain 5×10^5^ TCID_50_/mL of Akabane virus. The lane numbers correspond to the following specimens: Lane 1, 10 ^–1^ dilutions; Lane 2, 10 ^–2^ dilutions; Lane 3, 10 ^–3^ dilutions; Lane 4, 10 ^–4^ dilutions; Lane 5, 10 ^–5^ dilutions; Lane 6, 10 ^–6^ dilutions; Lane 7, 10 ^–7^ dilutions; Lane 8, 10 ^–8^ dilutions; Lane M, 100-bp DNA Ladder.

The results demonstrated that only Akabane virus was detectable by RT-LAMP assay (Figure 1). In contrast, this assay did not detect the other tested viral strains. Following *Acc*III digestion, the amplification products appeared as 2 fragments with sizes of 33 and 180 bp (Figure 2). The results also showed that the RT-LAMP assay is highly sensitive as it can detect a template at 10 ^–5^ dilution with a detection limit of 5.0 TCID_50_/mL (Figure 3). RT-LAMP exhibited high concordance with the semi-nested RT-PCR for Akabane virus detection in clinical samples (Table 1). Fifty-one out of 126 samples were positive for the Akabane virus using both RT-LAMP and semi-nested RT-PCR assays. One negative sample from semi-nested RT-PCR was positive by RT-LAMP assay, and 3 negative samples from RT-LAMP assay were positive by semi-nested RT-PCR. An additional seventy-one samples also were negative from both assays. Accordingly, the cohen’s kappa coefficient for these two assays was 96.62%.

**Table 1:** Comparative analysis of clinical samples for Akabane virus positivity by RT-LAMP assay and semi-nested RT-PCR

**RT-LAMP assay**	**Semi-nested RT-PCR**		
	**Positive**	**Negative**	**Total**
Positive	51	1	52 (41.27%)
Negative	3	71	74 (58.73%)
Total	54 (42.86%)	72 (57.14%)	126

In this study, we report a novel and rapid molecular detection technique named RT-LAMP assay to detect Akabane virus from clinical samples with high specificity (Figure 2). This assay can distinguish Akabane virus from other four viruses known to cause abortion in cattle (bovine herpesvirus 1, bovine parvovirus, bluetongue virus, and bovine viral diarrhea virus). In addition, the developed RT-LAMP is simple and rapid compared to other molecular detection assays (single RT-PCR, nested RT-PCR, or real-time RT-PCR). Therefore, RT-LAMP has great potential for use as a field test to detect Akabane viral infection.

**Figure 4:**
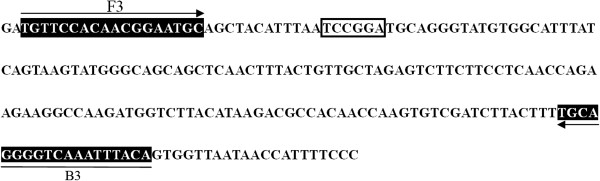
**Locations of RT-LAMP primers.** Oligonucleotide primers binding sequences are indicated by arrows. The *Acc*III restriction site is boxed.

A critical factor when performing the RT-LAMP assay is to select a conserved nucleic acid fragment for the design of specific primers. In the present study, 25 sequences of the nucleoprotein gene of Akabane virus reported in GenBank were analyzed, and a highly conserved fragment was chosen as the target region (Figure 4). Two pairs of specific primers (two inner primers and two outer primers) were designed using the Primer Explorer Version 4 software. On these bases, these primers were further analyzed by Blast N software. The rigorous selection and analysis of high conservation fragment are conducive to the specificity of established RT-LAMP.

The detection of positive amplification of RT-LAMP can be accomplished by agarose gel electrophoresis and real-time monitoring using a turbidimeter. In addition, amplification of the target gene can also be visualized by a fluorescent intercalating dye. In this study, SYBR Green I was used for detection of the amplified products by observing color change. The orange color of the dye will fluoresce green under natural light with positive amplification. For the cases with no amplification, the orange color of the dye is retained.

We show that the RT-LAMP assay using 126 clinical samples demonstrated that the RT-LAMP assay is high sensitive and accurate, similar to the semi-nested RT-PCR assay. The detection method also demonstrated that the Akabane virus infection is common in calves for various geographical areas of Xinjiang province. Thus, preventive measures are recommended for the cattle industry.

In conclusion, a novel RT-LAMP assay was successfully developed for the rapid detection of Akabane virus genomic RNA in clinical samples from abortive cases. The assay is highly sensitive and specific. This rapid, easy-to-operate, and sensitive assay can be used for the diagnosis and epidemiological surveillance of Akabane virus infection in resource-limited countries or regions.

## Materials and methods

### Collection of clinical samples

A total of 126 clinical samples were from aborted bovine fetuses from 8 cattle farms located in 6 geographical areas (Taichen, Kuitun, Yili, Shihezi, Manas, Shawan) of Xinjiang province, China during October 2008 through February 2011. These samples consisted of brain, muscles, spleen, kidney, heart, lung, and lymph nodes. Samples were kept cool by the staff of the Ili Kazakh Autonomous Prefecture Center of Animal Disease Control and Diagnosis and delivered on ice to key laboratory of the Preventive Veterinary Medicine, College of Animal Science, and Technology (Shihezi University) within 24–48 h.

### Viruses and cells

Akabane virus strain JaGAR 39/MB19 was originally supplied by Dr Inaba of the National Institute of Animal Health, Japan. BHK-21 cells were maintained in minimum essential medium (MEM, Invitrogen, Carlsbad, CA, USA) containing 10% fetal bovine serum (FBS, Biofluid, Richmond, VA, USA). Bovine herpesvirus 1 strain 758, bovine parvovirus strain BF15, bluetongue virus strain CSIRO 154, and bovine viral diarrhea virus strain NADL were provided by the State Key Lab of Veterinary Etiological Biology.

### Design of specific RT-LAMP primers

Nucleoprotein (N) gene sequences of 25 different strains/isolates of *Akabane virus* were obtained from GenBank, and the homology was analyzed using the molecular software DNAMAN. The conserved fragment with high homology was chosen to be the target region (Figure 4) which was used to design the *Akabane virus* RT-LAMP primers by the Primer Explorer V4 software (http://primerexplorer.jp/e/) (Table 2), as previously described by Notomi et al. [20].

**Table 2:** Oligonucleotide primers used for RT-LAMP assay and semi-nested RT-PCR against Akabane virus

**Primers**	**Sequence (5'-3')**	Nucleotide positions*	**Length (bp)**
F3	TGTTCCACAACGGAATGC	27–44	18
B3	TGTAAATTTGACCCCTGCA	198–216	19
FIP	GAGCTGCTGCCCATACTTACT-AGCTACATTTAATCCGGATGC		42
BIP	TACTGTTGCTAGAGTCTTCTTCCTC-AGTAAGATCGACACTTGGTT		45
N1	ATGTTCCACAACGGAATGCAGC	25–46	22
N2	AGCCAGGAAAGCTCTAGCTGCAG	680–658	23
N3	GGAAAATGGTTATTAACCACT	215–235	21

*Nucleotide sequence position according to nucleoprotein (N) gene of *Akabane virus* strain 93FMX (GenBank accession number FJ498797).

### RNA extraction

Total RNA was extracted from Akabane virus-infected BHK-21 cells or clinical samples with SV-total RNA Isolation System (Promega, Mannheim, Germany), according to the manufacturer’s protocol. The RNA was eluted in 20 μL of RNase-free water containing 0.04% sodium azide and was stored at -80°C until further use. The RNA concentration (ng/μL) was measured with a spectrophotometer.

### RT-LAMP

The RT-LAMP reaction was carried out in a 25 μL volume containing 12.5 μL of LAMP buffer (20 mM Tris–HCl [pH 8.8], 10 mM KCl, 8 mM MgSO_4_, 10 mM (NH_4_)_2_SO_4_, 0.1% Triton X-100, 0.8 M betaine, and 1.4 mM each of deoxynucleoside triphosphates), 2 μL of primer mixture (40 pM each of FIP and BIP, and 5 pM each of F3 and B3), 1 μL of *Bst* DNA polymerase (8 U), 1 μL of *Avian myeloblastosis virus* reverse transcriptase (40 U), 2 μL of template RNA, and 6.5 μL of distilled water. The reaction mixture was incubated at 63°C for 60 min in a heating block, followed by heating at 80°C for 2 min to terminate the reaction. After the reaction, a 10 μL aliquot of the RT-LAMP product was subjected to electrophoresis in 2.0% agarose gel and visualized by staining with 0.5 μg/mL of ethidium bromide under ultraviolet light. For visual fluorescence detection, 1 μL of a 10-fold dilution of SYBR Green I (1000×) was added into the reaction mixture, and the positive amplification was determined according to the color change (orange and green in a negative and positive reaction, respectively).

### Analytical sensitivity and specificity of RT-LAMP

To evaluate the specificity of the RT-LAMP reaction, four viruses known to cause abortion in cattle, *bovine herpesvirus* 1 (strain 758), *bovine parvovirus* (strain BF15), *bluetongue virus* (strain CSIRO 154) and *bovine viral diarrhea virus* (strain NADL) were used. To determine the sensitivity of RT-LAMP assay, the culture supernatant containing 5×10^5^ TCID_50_/mL of Akabane virus was diluted 10-fold and amplified by RT-LAMP assay. For further confirmation, the amplified products were confirmed by DNA sequencing and digested with restriction enzyme *Acc*III (Promega) and their sizes of digested products were analyzed by electrophoresis in 3% agarose gels. Visualization was performed by straining with ethidium bromide.

### Semi-nested RT-PCR

The semi-nested RT-PCR was performed using the specific primers N1 (outer sense primer), N2 (antisense primer) and N3 (inner antisense primer) (Table 2). The N1 and N2 primers were used for the first amplification after reverse transcription reaction, while N1 and N3 were used for the second round of amplification (semi-nested). Briefly, RT reactions were carried out with the SuperScript III Reverse Transcriptase kit (Invitrogen) following the manufacturers protocol. Two μL of template RNA was used to synthesize the first strand cDNA at 50°C for 30 min, followed by incubation at 94°C for 5 min. The reaction mixture for the first PCR step consisted of 2 μL of cDNA template, 250 nmol/L of each primer (N1 and N2 ), 4 mmol/L MgCl_2_, PCR buffer, 200 μmol/L of each deoxynucleoside triphosphate, 1.25 units of Taq DNA polymerase (TaKaRa Bio. Co., Ltd., Japan), and water to a final volume of 50 μL. The PCR was then carried out for 30 cycles at 94°C for 4 min; 30 cycles at 94°C for 30 s, annealing at 55°C for 30 s, extension at 72°C for 1 min; and final extension at 72°C for 10 min. 2 μL of the first amplification product was used as DNA template for each of the 25 μL secondary amplifications. The conditions and concentrations of the secondary amplification were identical to those of the primary, except for the primers (N1 and N3) and extension at 72°C for 30 s. The PCR products of the secondary amplification round were analyzed by 3% gel electrophoresis and stained with ethidium bromide, and confirmed by DNA sequencing.

### Evaluation of the RT-LAMP Using Clinical Samples

The clinical samples were subjected to total RNA extraction as described above. The RT-LAMP assay was used to detect the presence of Akabane virus. In addition, semi-nested RT-PCR was employed to examine the presence of Akabane virus and for testing the consistency between the two assays (RT-LAMP and semi-nested RT-PCR). The Cohen’s kappa coefficient between RT-LAMP and semi-nested RT-PCR was calculated according to the following formula.

### Ethical approval

The experiments were carried out in accordance with the guidelines issued by the Ethical Committee of Shihezi University.

## Abbreviations

RT-LAMP: Reverse transcription loop-mediated isothermal amplification; RT-PCR: Reverse transcription polymerase chain reaction; N: Nucleocapsid; TCID50: Median tissue culture infective dose; VNT: Virus neutralization test.

## Competing interests

The authors declare that they have no competing interests.

## Authors’ contributions

JQ and QLM conceived of the study, analyzed the results and drafted the manuscript; JWW supervised the research, analyzed the results and helped draft the manuscript; GCW, YCL, ZHH, HBY and ZCZ performed the experiments; XPC and CFC supervised the research and helped draft the manuscript. All authors contributed to write and revise the article critically for important intellectual content and have approved the final version of the manuscript to be published.
